# Bias of allele-sharing linkage statistics in the presence of intermarker linkage disequilibrium

**DOI:** 10.1186/1471-2156-6-S1-S82

**Published:** 2005-12-30

**Authors:** Ellen L Goode, Michael D Badzioch, Gail P Jarvik

**Affiliations:** 1 Cancer Prevention Program, Fred Hutchinson Cancer Research Center, Seattle, WA, USA; 2 Department of Health Sciences Research, Mayo Clinic College of Medicine, 200 First Street SW, Rochester, MN 55905 USA; 3 Division of Medical Genetics, University of Washington, Seattle, WA, USA; 4 Department of Genome Sciences, University of Washington, Seattle, WA, USA

## Abstract

Current genome-wide linkage-mapping single-nucleotide polymorphism (SNP) panels with densities of 0.3 cM are likely to have increased intermarker linkage disequilibrium (LD) compared to 5-cM microsatellite panels. The resulting difference in haplotype frequencies versus that predicted may affect multipoint linkage analysis with ungenotyped founders; a common haplotype may be assumed to be rare, leading to inflation of identical-by-descent (IBD) allele-sharing estimates and evidence for linkage. Using data simulated for the Genetic Analysis Workshop 14, we assessed bias in allele-sharing measures and nonparametric linkage (NPL_all_) and Kong and Cox LOD (KC-LOD) scores in a targeted analysis of regions with and without LD and with and without genes. Using over 100 replicates, we found that if founders were not genotyped, multipoint IBD estimates and *δ *parameters were modestly inflated and NPL_all _and KC-LOD scores were biased upwards in the region with LD and no gene; rather than centering on the null, the mean NPL_all _and KC-LOD scores were 0.51 ± 0.91 and 0.19 ± 0.38, respectively. Reduction of LD by dropping markers reduced this upward bias. These trends were not seen in the non-LD region with no gene. In regions with genes (with and without LD), a slight loss in power with dropping markers was suggested. These results indicate that LD should be considered in dense scans; removal of markers in LD may reduce false-positive results although information may also be lost. Methods to address LD in a high-throughput manner are needed for efficient, robust genomic scans with dense SNPs.

## Background

Gene-mapping endeavors currently assess linkage of up to 11,555 single-nucleotide polymorphisms (SNPs) distributed throughout the genome [1]. Increased marker density of these maps over 5-cM microsatellite maps is likely to result in increased intermarker linkage disequilibrium (LD). Thus, observed haplotype frequencies may differ from that computed from individual marker allele frequencies.

Marker allele frequencies are used in linkage analysis for the estimation of missing genotypes probabilities. For two-point linkage analysis, over or underestimation of allele frequencies may lead to false-positive results [2]; a common allele may be assumed to be rare, leading to inflation in probability of being shared identically by descent (IBD). It follows that in multipoint analyses, over- or underestimation of haplotype frequencies may also influence validity of linkage results [3]; a common haplotype may be assumed to be rare, leading to inflation in IBD allele-sharing. Most multipoint linkage methods rely on the assumption of intermarker linkage equilibrium.

The density of currently available SNP maps (0.31 cM) [1] is similar to the average density of markers in the simulated data provided for Genetic Analysis Workshop 14 (GAW14) (0.29 cM). We sought to assess whether intermarker LD affected bias of nonparametric linkage (NPL) statistics by performing targeted analyses before and after LD reduction in regions with and without simulated LD and with and without simulated genes.

## Methods

### Population and phenotypes

The Aipotu population of 100 nuclear families simulated for GAW14 was used because of its relatively high prevalence of the phenotypes studied. One hundred replicates were separately analyzed. Analyses were performed with and without founder genotypes. Two dichotomous traits were analyzed: Trait H, due to Gene D2 in a region with LD, and Trait B, due to Gene D1 in a region without LD. Both traits were monogenic, dominant, and had no phenocopies. Penetrance and prevalence were 20% and 7.4% for Trait H and 30% and 2.1% for Trait B. All analyses were performed with full knowledge of the simulated genetic models [4].

### Chromosomal regions

**Figure 1:**
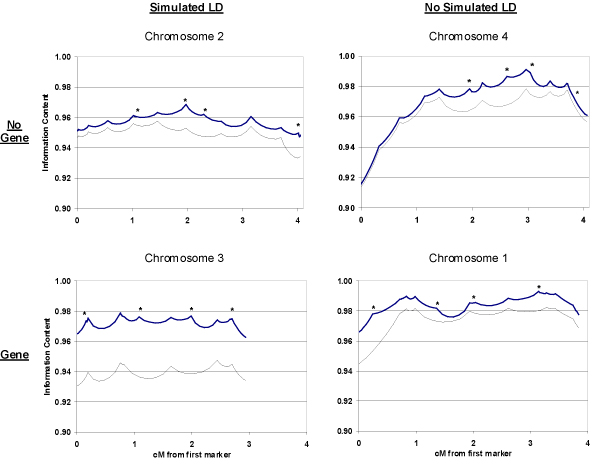
**Chromosomal regions analyzed**. Thick line, LD not reduced; thin line, LD reduced; *, marker dropped to reduce LD

Four chromosomal regions were analyzed (Figure 1). A region with simulated LD and no genes on chromosome 2 between B02T1014 and B02T1028 (4.36–8.31 cM) was analyzed for assessment of false-positive results. A region with simulated LD and the gene D2 on chromosome 3 was analyzed to assess LD effects on power; LD extended from B03T3056 (296.39 cM) to gene D2 (just after B03T3067, 299.32 cM).

Two regions without simulated intermarker LD were analyzed (Figure 1). These regions were a non-gene region on chromosome 4 between B04T3485 and B04T3499 (119.24 – 123.31 cM), and the region with gene D1 on chromosome 1 between B01T0554 and B01T0567 (167.00 – 170.84 cM). These regions were used because of similar marker density as the two LD regions. Thick lines graphed in Figure 1 represent multipoint information content (IC) in each region.

### LD assessment and reduction

LDMAX [5] and GOLD [5] were used to calculate and display pairwise |D'| and *r*^2 ^values based on the estimation maximization of founder haplotype frequencies in the second Aipotu replicate [6]. One megabase was assumed to approximate 1 cM. LD was reduced by dropping alternate SNPs in pairs with |D'| > 0.73; this cut-point was chosen so that an equal number of markers were dropped in gene and non-gene regions. SNPs were dropped which created the shortest gaps.

### Allele-sharing measures and linkage statistics

Multipoint NPL_all _scores and Kong and Cox (KC)-LOD scores were calculated for each replicate using MERLIN v. 0.10.2 [7] which implements a sparse binary tree extension to the Lander-Green algorithm [8]. Both statistics assess the IBD allele-sharing among affected relatives. NPL_all _scores are normalizations of scores based on observed phenotypes and the binary inheritance vector at each location [9,10]. KC-LOD scores are based on *δ*, the free parameter in a one-parameter allele-sharing model; under the null, *δ *equals 0, and, under the alternative, *δ *is greater than 0 [11]. *θ *was converted to centimorgans using the Kosambi map function.

We compared regions with and without LD, and we compared regions with LD before and after LD reduction. We performed analyses under a variety of conditions: 1) whether allele frequencies were estimated from all individuals or from founders and 2) whether linkage statistics were calculated at five evenly spaced intervals between markers or at 0.2-cM intervals.

For each replicate (*n *= 100), the mean probability of sharing 0, 1, and 2 alleles IBD across markers and across relative pairs was determined, and the mean value of  and the mean NPL_all _and KC-LOD scores (and their corresponding *p*-values) across markers pairs was determined. These statistics (prob(0), prob(1), and prob(2), , NPL_all _and *p*-value, KC-LOD and *p*-value) were then summarized across all replicates.

## Results

One hundred replicates of the 100 Aipotu families were analyzed separately for Trait H (chromosome 2 and 3) and Trait B (chromosome 4 and 1). On average, each replicate contained 229 sibling pairs affected with Trait H and 119 sibling pairs affected with Trait B.

### LD assessment and reduction

**Figure 2:**
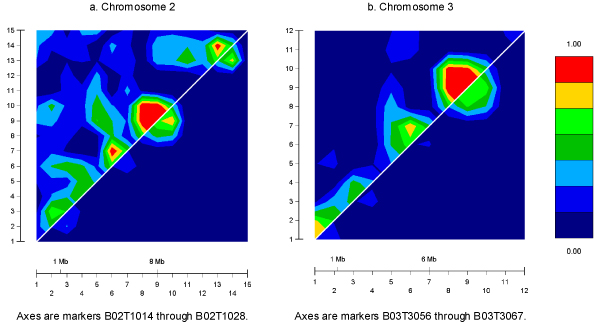
**Pairwise disequilibrium coefficients: simulated LD present. **|D'| above and *r*^2 ^below diagonal

**Figure 3:**
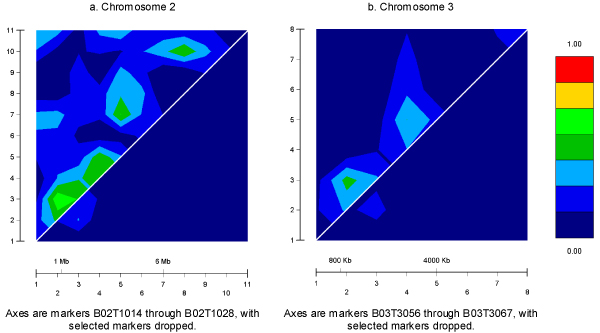
**Pairwise disequilibrium coefficients: simulated LD reduced. **|D'| above and *r*^2 ^below diagonal

LD was assessed among founders in the four regions. As expected, intermarker LD was observed on chromosomes 2 and 3 (Figure 2) and not on chromosomes 1 and 4. To reduce LD, genotypes were dropped at correlated markers with |D'| greater than 0.73 (see Methods). On chromosome 2, dropping C02R0094, B02T1021, B02T1023, and B02T1027 (markers 6, 8, 10, and 14) reduced LD to this level (Figure 3a). On chromosomes 3, dropping B03T3057, B03T3061, B03T3063, and B03T3065 (markers 2, 6, 8, and 10) reduced LD, such that the maximum |D'| was 0.49 (Figure 3b). B04T3490, B04T3492, B04T3494, C04R0321 B01T0555, B01T0559, B01T0561, and B01T0563 were dropped in the non-LD regions of chromosomes 4 and 1. Thin lines in Figure 1 show the decrease in IC when markers were dropped. Mean IC decreased by 1% for chromosomes 2, 4, and 1 and 3% for chromosome 3.

### Allele-sharing measures

There was a modest increase in estimated allele-sharing in the region with LD and without a gene on chromosome 2 when founders were ungenotyped; prob(2) increased slightly from 0.336 ± 0.468 with founders to 0.342 ± 0.471 without founders. The non-gene region without simulated LD on chromosome 4 did not show any increase in allele-sharing with ungenotyped founders. Reduction of LD in the region with simulated LD reduced the upward bias in IBD allele-sharing (prob(2) = 0.340 ± 0.469), suggesting that the bias may be due to LD.

Estimated *δ *parameters are provided in Table 1. When founders were genotyped, the distributions were as expected based on simulation;  was elevated when a gene was present and centered on null otherwise. However, when founders were not genotyped, inflation in  was seen in the chromosome 2 region with LD and no gene (mean  = 0.06 ± 0.10). This was not seen in the in chromosome 4 region with no LD and no gene (mean  = 0.00 ± 0.11). Reduction of LD brought  slightly closer to null on chromosome 2 (mean  = 0.04 ± 0.10), consistent with LD being the reason for the observed upward bias.

### Linkage statistics

When founders were genotyped and all markers were used, results were as expected based on simulation parameters (Table 2). After LD was reduced, evidence for linkage was slightly reduced for regions with genes. This loss in power was expected because true linkage information was removed when linked markers were dropped (Figure 1).

With ungenotyped founders, an upward bias in NPL_all _and KC-LOD scores was observed in the region with no gene but with LD on chromosome 2 (Table 2). Mean NPL_all _and KC-LOD scores were inflated from null to 0.51 and 0.19, respectively. The region with no gene and no LD did not show this inflation of linkage statistics. These results suggest that the inflation may be due to increased LD. In addition, reduction of LD on chromosome 2 brought the mean NPL_all _and KC-LOD scores closer to null (0.36 and 0.14, respectively). No differences in results were seen in the region without LD and without a gene (chromosome 4) when markers were removed. In the regions with genes, again, a reduction in power with dropping of markers was observed.

Comparison of the *p*-value distributions for regions without genes (simulated null distributions) also suggested an upward bias in the presence of LD. On chromosome 2 with simulated LD, the fifth percentile *p*-values for NPL_all _and KC-LOD scores were 0.06 and 0.06, respectively. When founders were not genotyped, these values decreased to 0.02 and 0.01, respectively, suggesting an increase in type I error. When LD was reduced, these values became 0.03 and 0.02, respectively. This trend was not seen on chromosome 4 without simulated LD.

Results were similar when calculated on a grid, rather than evenly spaced between markers, and when allele frequencies were estimated from the dataset, rather than founders.

## Discussion

Our results suggest that reduction of intermarker LD may reduce false-positive rates (improve the validity) of NPL_all _and KC-LOD scores via reducing overestimation of IBD when founders are not genotyped. In studies of late-onset diseases, pedigree founders are often not available and marker allele frequencies are required. It has been shown that, for two-point analysis, errors in marker allele frequencies may lead to false-positive results when a common marker is assumed to be rare [2]. Because LD creates unexpected haplotype frequencies, a similar false-positive multipoint result without founders may be possible.

This analysis has several limitations. Only 100 replicates were examined, and analyses were performed under a limited configuration of parameters. We examined effects of LD on mean NPL_all _and KC-LOD scores across regions and did not consider width of linkage peaks. We considered only nuclear families, but expect results to be similar with allele-sharing methods in extended pedigrees. We did not consider traditional LOD scores although these may be susceptible to inflated type I error rates as well [12]. We also did not assess effects of LD between markers and disease which may result in loss of power and underestimation of *θ *[13].

Issues arise in attempting to account for LD in linkage analysis using the methods described here. First, choice of an LD coefficient and its cut-off or other test for its significance will affect regions to be addressed. Although we removed |D'| greater than 0.73, this could be varied to optimize the balance between bias and informativeness. Second, specific markers to drop in an LD region must be selected. We dropped markers such that shorter map gaps were created; an alternative is to choose based on IC, as proved useful in a recent empirical report [14].

Dropping markers in LD in the current analysis appeared to reduce power in areas with true linkage. This is an important loss, because, in reality one can not differentiate true and false positives. Software allowing for estimation and/or fixing of haplotype-frequencies in LOD score linkage analysis without dropping markers was developed for early restriction fragment length polymorphism studies (described in [15]). However, implementation over genome-wide high-density SNPs will be cumbersome. High-throughput methods for parametric and nonparametric linkage analyses accounting for population-specific intermarker LD in genomic searches without reduction of IC are needed.

## Conclusion

As linkage analyses are conducted on dense SNP genome scans, one issue to weigh will be increased intermarker LD over microsatellite genome scans. Genome-wide analysis of LD should be performed preliminarily so that LD can be accounted for and bias away from the null can be minimized. Simple methods to account for LD, such as marker-dropping, or more sophisticated analytical approaches may improve validity of these types of linkage studies.

## Abbreviations

GAW14: Genetic Analysis Workshop 14

IBD: Identical by descent

IC: Information content

KC-LOD: Kong and Cox LOD

LD: Linkage disequilibrium

NPL: Nonparametric linkage

SNP: Single nucleotide polymorphism

## Authors' contributions

ELG designed the study, performed analyses, and wrote the manuscript. MDB provided critical input on analyses and manuscript. GPJ guided analyses and edited the manuscript.

## Figures and Tables

**Table 1:** Estimated delta parameters in the presence and absence of LD

	Simulated			Founders genotyped	Founders ungenotyped
				
Chromosome	Gene	LD	LD reduction	Mean ± SD	Mean ± SD
2	No	Yes	Not reduced	0.00 ± 0.09	0.06 ± 0.10
			Reduced	0.00 ± 0.09	0.04 ± 0.10
4	No	No	Not reduced	0.00 ± 0.12	0.00 ± 0.11
			Reduced	0.00 ± 0.12	0.00 ± 0.12
3	Yes	Yes	Not reduced	0.36 ± 0.09	0.38 ± 0.09
			Reduced	0.36 ± 0.09	0.38 ± 0.10
1	Yes	No	Not reduced	0.48 ± 0.09	0.46 ± 0.09
			Reduced	0.48 ± 0.09	0.47 ± 0.09

100 replicates, allele frequencies from file, statistics calculated at five evenly spaced intervals between markers.

**Table 2:** NPL statistics in the presence and absence of LD

	Simulated			Founders genotyped		Founders ungenotyped	
				
Chromosome	Gene	LD	LD reduction	Mean NPL_all _± SD (Range)	Mean KC-LOD ± SD (Range)	Mean NPL_all _± SD (Range)	Mean KC-LOD ± SD (Range)
2	No	Yes	Not Reduced	0.01 ± 0.93 (-2.28, 2.96)	0.01 ± 0.33 (-1.17, 1.72)	0.51 ± 0.91 (-1.57, 3.15)	0.19 ± 0.38 (-0.68, 2.02)
			Reduced	0.00 ± 0.92 (-2.29, 2.86)	0.00 ± 0.33 (-1.24, 1.64)	0.36 ± 0.90 (-1.82, 2.79)	0.14 ± 0.35 (-0.77, 1.60)
4	No	No	Not Reduced	-0.01 ± 0.92 (-2.81, 2.26)	-0.01 ± 0.30 (-1.32, 1.24)	-0.02 ± 0.82 (-2.77, 2.21)	0.00 ± 0.28 (-1.35, 1.27)
			Reduced	-0.03 ± 0.93 (-2.80, 2.23)	-0.01 ± 0.31 (-1.32, 1.26)	-0.04 ± 0.83 (-2.68, 2.18)	-0.01 ± 0.29 (-1.31, 1.32)
3	Yes	Yes	Not Reduced	3.89 ± 1.03 (1.64, 6.69)	3.22 ± 1.64 (0.56, 9.18)	3.72 ± 0.98 (1.72, 6.46)	3.25 ± 1.61 (0.58, 8.68)
			Reduced	3.84 ± 1.04 (1.64, 6.73)	3.20 ± 1.68 (0.57, 9.44)	3.46 ± 1.00 (1.50, 6.24)	3.03 ± 1.62 (0.48, 8.31)
1	Yes	No	Not Reduced	4.61 ± 1.05 (2.15, 8.51)	4.06 ± 1.80 (0.88, 11.79)	3.79 ± 0.95 (1.73, 7.18)	3.22 ± 1.58 (0.70, 9.52)
			Reduced	4.58 ± 1.03 (2.13, 8.42)	4.05 ± 1.77 (0.87, 11.67)	3.71 ± 0.93 (1.32, 6.89)	3.19 ± 1.53 (0.42, 9.06)

100 replicates, allele frequencies from file, statistics calculated at five evenly spaced intervals between markers.
